# Surgical Treatment of Axillary Hidradenitis Suppurativa Using Preliminary Defect Closure and Two Sliding Island Triangular Skin-Subcutaneous Flaps from the Arm and Chest

**DOI:** 10.3390/jcm14175945

**Published:** 2025-08-22

**Authors:** Andrzej Bieniek, Filip Majda, Iwona Chlebicka, Klaudia Knecht-Gurwin, Jacek C. Szepietowski

**Affiliations:** 1 University Center for General and Oncological Dermatology, Wroclaw Medical University, 50-367 Wroclaw, Poland; andrzej.bieniek@umw.edu.pl (A.B.); klaudia.knecht@gmail.com (K.K.-G.); 2 Regional Specialist Health Care Center—Diabetology Clinic, Dobrzyńska 21/23, 50-403 Wroclaw, Poland; filip_majda@interia.pl; 3 Department of Dermato-Venereology, 4th Military Hospital, 50-981 Wroclaw, Poland; iwonak4wsk@interia.pl; 4 Division of Dermatology, Venereology and Clinical Immunology, Faculty of Medicine, Wroclaw University of Science and Technology, 50-377 Wroclaw, Poland

**Keywords:** hidradenitis suppurativa, star technique, triangular flap, surgery

## Abstract

**Background**: Surgical excision of axillary hidradenitis suppurativa (HS) often results in challenging defects. While flap techniques offer durable coverage, they are typically invasive. We present a modified method combining peripheral defect reduction with two sliding triangular island flaps from the arm and chest, designed to optimize healing with minimal invasiveness. **Methods:** Twelve patients (Hurley II–III) underwent excision and dual V-Y advancement flap reconstruction. Flaps were mobilized without perforator dissection. Outcomes were assessed using patient and surgeon Likert-scale ratings at one and six months. **Results:** Good healing was achieved in 91.7% of cases. Both patient acceptance and cosmetic outcomes were favorable (83.3%). No major complications were noted; one recurrence (8.3%) occurred at six months. **Conclusions:** The described technique offers a safe, effective, and cosmetically favorable option for medium-sized axillary HS defects. It provides a less invasive alternative to conventional flaps, with high healing rates and patient acceptance.

## 1. Introduction

Hidradenitis suppurativa (HS) is a chronic, recurrent, and often debilitating inflammatory skin disorder that predominantly affects intertriginous areas such as the axillae, groin, and perianal region. The disease imposes a substantial burden on patients, not only due to pain, malodorous discharge, and functional limitations, but also because of its psychological and social impact. In more advanced stages, particularly Hurley stage II and III, medical therapies are often insufficient to achieve sustained remission, and surgical intervention becomes a key component of management [1].

Surgical excision is widely recognized as an effective method for removing affected tissue and reducing recurrence rates in moderate to severe HS. Procedures are performed in various anatomical locations, with the axillary region being among the most frequently treated sites. One of the main consequences of surgical treatment is the formation of large postoperative defects, necessitating appropriate reconstructive strategies. These should support optimal wound healing, minimize the risk of complications, and ensure satisfactory functional and cosmetic outcomes. Over the years, a range of reconstructive methods has been developed, including healing by secondary intention, simple closure, split-thickness skin grafts, and various flap techniques [1,2,3,4]. Combined approaches—such as partial closure followed by secondary healing or skin grafting—have also been employed [1]. However, each of these options has specific limitations. Secondary healing can be prolonged and uncomfortable, skin grafts often lead to unaesthetic scarring and contractures, and flap reconstructions, though effective, are frequently invasive and technically demanding. Based on the authors’ experience gathered from several hundred HS surgeries, simple closure is a viable option for small to medium-sized defects. However, extensive wounds often pose significant challenges: primary closure in such cases may lead to wound dehiscence, poor adhesion of tissue to the wound bed, and elevation of wound edges, all of which delay healing. Therefore, in our clinical practice, simple closure is seldom used for medium-sized axillary defects. Instead, we more frequently rely on spontaneous healing, often preceded by partial defect reduction using the so-called “star technique.” This method involves excising four to five small triangular skin segments from the wound periphery and suturing the resulting gaps, thereby reducing the surface area of the open wound and accelerating granulation [1]. This approach reflects a pragmatic balance between technical feasibility, tissue preservation, and the need for efficient and cosmetically acceptable wound closure. To further optimize outcomes, we developed a modified version of this technique in 2020, which is presented in detail below.

## 2. Materials and Methods

**Figure 1:**
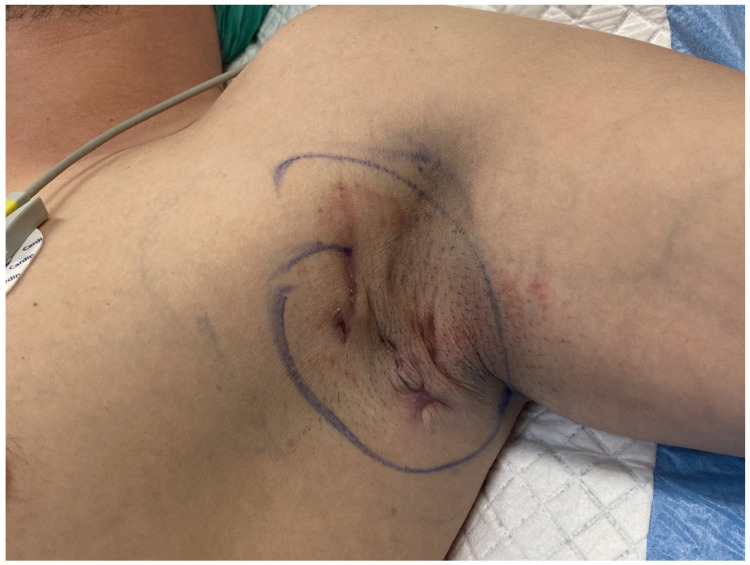
Preoperative view of the left axilla in a 36-year-old male patient.

**Figure 2:**
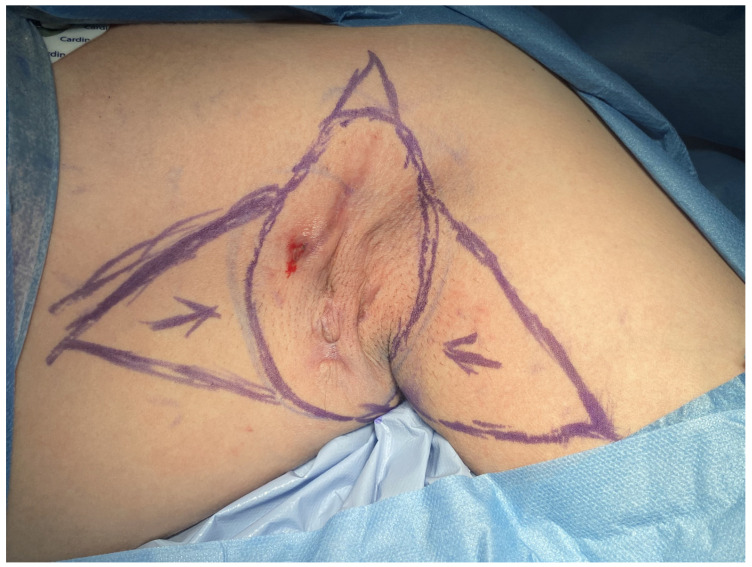
Marked incision lines outlining the resection area and flap design.

**Figure 3:**
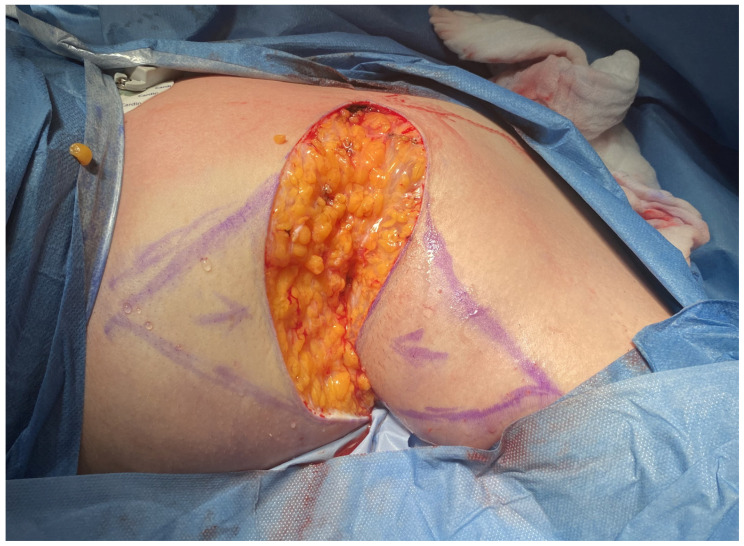
Post-excision wound with removal of peripheral upper and lower triangular skin segments.

**Figure 4:**
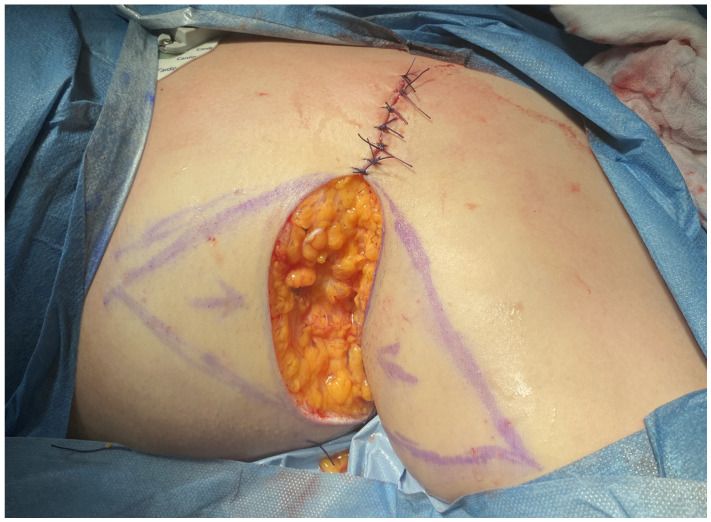
Partial primary closure of the defect along superior and inferior borders.

**Figure 5:**
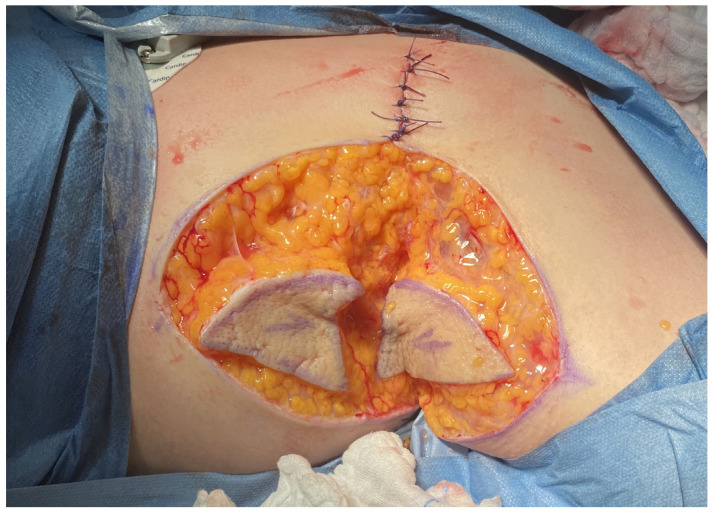
Mobilization of two V-Y advancement triangular skin-fat flaps—one from the arm and one from the lateral chest—advanced toward the wound center.

**Figure 6:**
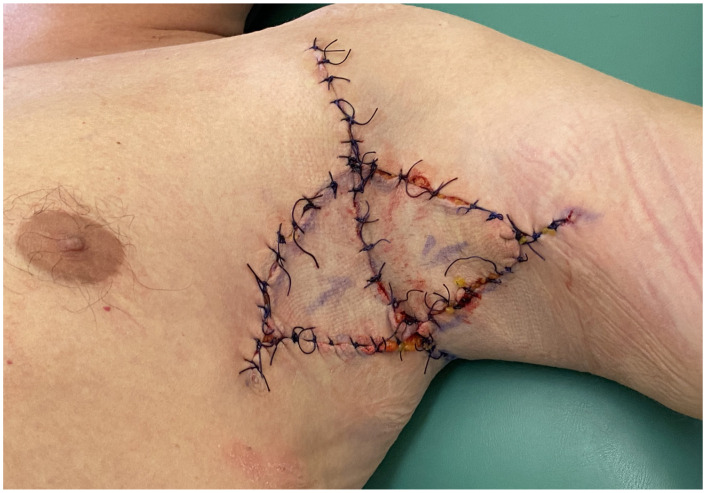
Final closure with wide simple sutures and application of a protective absorbent dressing.

**Figure 7:**
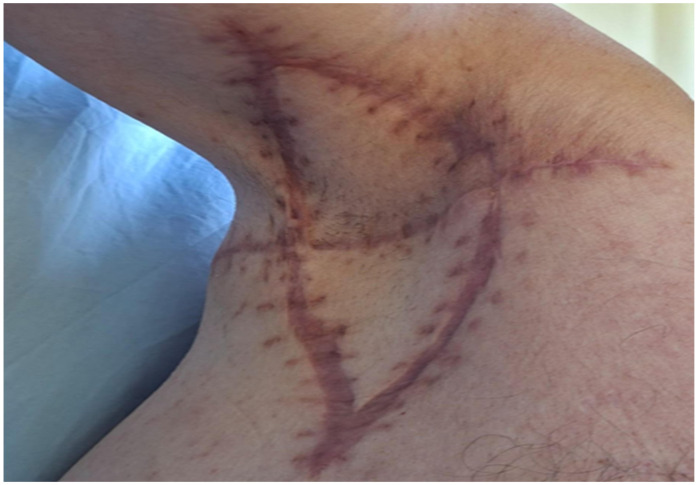
Six-month postoperative view showing complete epithelialization of the surgical site with postoperative scar formation.

From 2020 to 2024, the proposed method, in accordance with the 1964 Helsinki Declaration, was performed on 12 patients. Ten patients underwent surgery on both the right and left axillae, while two patients had surgery only on the right axilla. All procedures were performed under general anesthesia as well as tumescent local anesthesia, after obtaining informed consent from the patients. This surgical approach was specifically developed to reduce procedural invasiveness while facilitating effective closure of medium-sized axillary defects. It comprises a series of coordinated operative steps, including radical excision of diseased tissue, reduction of peripheral wound margins, and final reconstruction using two triangular skin-subcutaneous island flaps harvested from the medial aspect of the arm and the lateral thoracic wall. Initial surgical planning involved meticulous clinical evaluation of the affected region. Figure 1 illustrates the preoperative condition of the axilla in the male patient, highlighting the extent of chronic inflammation and dermal remodeling prior to intervention. Following antiseptic skin preparation, precise incision lines were delineated to define the resection margins and the geometric configuration of the intended advancement flaps. These intraoperative markings served as a critical anatomical roadmap for the subsequent surgical stages (Figure 2). Radical excision of all pathologic tissue was then undertaken, encompassing removal of diseased skin, fibrotic subcutaneous tissue, and any associated sinus tracts. In addition, small triangular cutaneous segments were excised from the superior and inferior wound peripheries to further reduce the surface area of the residual defect. This resulted in an oval-shaped post-excisional wound bed, adequately prepared for staged closure (Figure 3). Subsequently, the upper and lower wound borders were approximated using simple interrupted sutures. This maneuver achieved substantial reduction of the central defect and optimized conditions for subsequent tissue advancement (Figure 4). Furthermore, partial closure at this stage mitigated postoperative tension and promoted more favorable wound healing dynamics. Thereafter, two triangular skin-subcutaneous island flaps were simultaneously mobilized—one from the medial aspect of the upper arm and the other from the lateral thoracic wall. Each flap was carefully incised and elevated together with its underlying adipose tissue. The flaps were then advanced toward the center of the defect using a V-Y advancement technique to achieve a tension-free approximation (Figure 5). Following successful flap mobilization, the final configuration of the closure was achieved by securing the approximated tissue margins. The completed reconstruction is shown in Figure 6, demonstrating centrally coapted flaps and stable wound closure. Figure 7 demonstrates the six-month postoperative outcome, showing complete wound closure.

Assessment of surgical quality was performed by the operating surgeon using a 5-point Likert scale focused on key intraoperative parameters, including surgical exposure, procedural difficulty, duration, intraoperative complications, technical outcome, and overall satisfaction. These evaluations were completed intraoperatively and immediately postoperatively on the day of surgery and repeated at postoperative day 7 to monitor early wound healing and procedural outcome. The questionnaire used for these assessments is presented in Appendix A.

Postoperative outcomes were evaluated at standardized follow-up visits at one and six months after surgery and included wound healing status, patient satisfaction, cosmetic appearance, scar tenderness, and recurrence. “Good healing” was defined as complete epithelialization of the surgical wound without infection, dehiscence, or signs of delayed granulation. “Patient satisfaction” was assessed using a separate 5-point Likert scale completed independently by each patient. “Good cosmetic result” referred to the surgeon’s assessment of the final scar and surrounding tissue, including symmetry, contour, and pigmentation, and was rated on a 5-point Likert scale; responses of 4 or 5 were categorized as good. “Scar tenderness” was determined through direct questioning of the patient regarding pain or discomfort on palpation or movement of the axilla; responses were binary (yes/no). “Recurrence” was defined as the appearance of new inflammatory nodules, abscesses, or draining sinus tracts within the operated site. The above parameters are summarized in Table 1.

## 3. Results

All patients in the study group were male, aged 21 to 53 years (mean age: 32 ± 12.1 years). Additional demographic and clinical characteristics of the study group are as follows: four out of twelve patients (33.3%) were receiving biologic therapy with adalimumab at the time of surgery. Four patients (33.3%) were obese and had diagnosed hyperlipidemia. Eight patients (66.7%) were active smokers. No other significant comorbidities were reported. In fifty percent of the patients, HS were classified as Hurley stage II, and the remaining 50% as Hurley stage III. The lesion area ranged from 46 to 96 cm^2^, with a mean size of 59 ± 15.61 cm^2^. The hospital stay ranged from one to three days. The results of the procedures are presented in Table 1.

According to the surgeon-completed questionnaire (Appendix A), surgical exposure and technical outcome were rated as “strongly satisfactory” (score 5) in 10 out of 12 cases (83.3%), with the remaining 2 cases rated as “somewhat satisfactory” (score 4). No intraoperative complications requiring intervention were reported. The duration of procedures was rated as “as planned” or “somewhat shorter than planned” in all cases.

## 4. Discussion

Small and medium-sized axillary wounds are most commonly closed using direct suturing. For larger defects, many authors consider split-thickness skin grafts the preferred method. However, these grafts are associated with a significant risk of healing disturbances, unaesthetic scarring, and contractures, and are therefore frequently criticized [5]. Flap-based reconstruction offers a thicker and more elastic skin cover and can be performed using various types of flaps, such as skin-fat, skin-fascia, or skin-muscle flaps. These can be either random-pattern (supplied by the subdermal plexus) or axial-pattern (based on anatomically defined vascular pedicles) and transferred as pedicled or free flaps. Nevertheless, these techniques are often complex, require extensive tissue dissection, carry a risk of damaging adjacent anatomical structures, and are associated with the potential for flap necrosis. Furthermore, their application is typically limited to defects of specific sizes.

Sliding V-Y triangular skin-subcutaneous flaps for the coverage of oval defects have been used since the 1960s, with early concepts dating back to the 19th century [6,7]. Their blood supply is derived either from small, randomly distributed subcutaneous vessels or from larger perforating vessels. The lateral thoracic region and the medial surface of the arm are both rich in such perforators. Key vessels in the lateral thoracic region include branches of the lateral thoracic artery, the pectoral branch of the thoracoacromial artery, and the thoracodorsal artery (a branch of the subscapular artery) [8]. In the medial arm region, important perforators originate from the brachial artery, its branches to the biceps muscle, and the superior ulnar collateral artery. As a result, flaps in these regions can be supported either by anatomically defined vascular pedicles or by randomly distributed vessels.

Preoperative localization of major perforators can be accomplished using anatomical mapping, Doppler ultrasonography, or intraoperative vascular identification [9,10]. In cases where flap mobility is adequate after preparation, precise vascular dissection may not be necessary—this is often true for longitudinally advanced flaps [5,8]. In our series, most cases did not require explicit perforator dissection, suggesting that flap survival relied on random vascular supply. However, in situations with limited mobility, identification and preservation of a specific perforator may be required, especially when using propeller flaps rotating around a single vascular pedicle [11,12].

A literature review revealed that similar V-Y advancement flaps for axillary reconstruction have been described by other authors, typically using elongated flaps with widths equal to that of the defect [8,13]. Our modification utilizes equilateral triangular flaps, each with a base approximately 50% of the defect width. This is made feasible by the prior reduction in wound size through primary closure of the peripheral triangles, with flap reconstruction used as the final closure step. By limiting the area of dissection, this method appears to be less invasive than classic flap-based approaches. As with the previously described “star technique,” our approach combines simple suturing and flap reconstruction (Bieniek–star technique) [1].

The relatively small size of each flap necessitates the use of two triangular flaps per wound in all cases. Compared to single triangular flaps described by other authors [8,13], our method keeps the scars closer to the axilla, which improves cosmetic outcomes and helps ensure more even tissue tension distribution. While some studies have described bilateral triangular flaps advanced in the anterior–posterior direction [14], we believe that harvesting the flaps from the arm and chest provides superior vascularization due to the richer perforator network in these areas.

For wound closure in HS, we use large, widely spaced, single-layer skin sutures. This technique helps minimize infection risk, facilitates early hematoma detection, and allows efficient postoperative drainage. It may reduce the risk of complications compared to standard perforator flap procedures, which show a 15% complication rate according to Vaillant et al. [15]. Compared to other flap-based techniques, the Bieniek–star method offers several notable advantages. It avoids deep dissection and the need for vascular pedicle isolation, thereby reducing operative complexity and the risk of intraoperative injury. Unlike classic musculocutaneous or perforator flaps, our approach limits the extent of undermining while still providing sufficient coverage and tension-free closure for medium-sized defects. Moreover, in contrast to primary closure, which is often feasible only in small wounds and associated with high tension and risk of dehiscence, the combination of peripheral reduction and dual-flap advancement ensures both functional and aesthetic benefits. This may be particularly valuable in the axillary region, where contracture and scarring can significantly impact mobility and quality of life.

Additionally, accumulating evidence supports the integration of surgical and medical management in patients with HS. In a multicenter randomized trial, Bechara et al. [16] demonstrated that combining wide excision with adalimumab significantly improved outcomes compared to surgery alone, with 48% of patients achieving HiSCR50 at week 12 versus 34% in the placebo group (*p* = 0.049). Importantly, the use of adalimumab was not associated with an increased risk of postoperative wound infection, complication, or hemorrhage. Offidani et al. [17] provided practical recommendations supporting the continuation of biologic therapy such as adalimumab or secukinumab during the perioperative period, emphasizing that combined medical–surgical strategies may enhance outcomes in patients with HS. In an observational study conducted by Salvador-Rodriguez et al. [18], patients receiving TNF-α inhibitors had milder recurrences, though healing was slower—likely due to more severe baseline disease. Moreover, DeFazio et al. [19] reported that in patients undergoing radical surgical excision, continuation of biologic therapy (infliximab or ustekinumab) led to lower recurrence and disease progression rates, with new lesions developing in only 18% of cases during follow-up. These findings support a multimodal therapeutic approach and emphasize the importance of close collaboration between dermatologists and surgeons to optimize long-term outcomes in patients with moderate to severe HS.

This study has several important limitations. First, the sample size was relatively small, comprising only 12 patients, which may limit the generalizability of the findings. Second, all participants in the study were male, precluding conclusions about the applicability of the technique in female patients. Third, the follow-up period was limited to six months, which may not be sufficient to fully capture late complications or recurrences. Lastly, the surgical outcomes were assessed by the operating surgeons themselves, which introduces a potential observer bias in the evaluation of healing quality, cosmetic results, and overall satisfaction.

## 5. Conclusions

The technique described—combining preliminary defect closure with two sliding triangular island skin-subcutaneous flaps harvested from the arm and chest—facilitates rapid wound healing with low complication rates and high patient satisfaction. This method can be safely and effectively applied to medium-sized axillary defects, including those involving complete excision of hair-bearing skin. It appears to reduce the invasiveness of flap surgery while improving safety and aesthetic outcomes.

## Figures and Tables

**Table 1:** Postoperative outcomes at one-month and six-month follow-up after axillary HS surgery using the Bieniek–star technique.

Outcome	1-Month Follow-Up (*n* = 12)	6-Month Follow-Up (*n* = 12)
Good healing	11/12 (91.7%)	11/12 (91.7%)
Bleeding	0/12 (0%)	0/12 (0%)
Patient satisfaction	10/12 (83.3%)	10/12 (83.3%)
Good cosmetic result (surgeon-assessed)	10/12 (83.3%)	10/12 (83.3%)
Scar tenderness	3/12 (25%)	3/12 (25%)
Recurrence	0/12 (0%)	1/12 (8.3%)

## Data Availability

The data underlying this article will be shared on reasonable request to the corresponding author.

## Appendix A

### A Questionnaire for Intraoperative and Postoperative Asssessment

1. Quality of Surgical Exposure:
  - 1—Strongly unsatisfactory
  - 2—Somewhat unsatisfactory
  - 3—Neutral
  - 4—Somewhat satisfactory
  - 5—Strongly satisfactory
2. Technical Difficulty of the Procedure:
  - 1—Strongly difficult
  - 2—Somewhat difficult
  - 3—Neutral
  - 4—Somewhat easy
  - 5—Strongly easy
3. Duration of the Procedure (compared to planned time):
  - 1—Strongly exceeded planned time
  - 2—Somewhat exceeded planned time
  - 3—As planned
  - 4—Somewhat shorter than planned
  - 5—Strongly shorter than planned
4. Intraoperative Complications:
  - 1—Multiple complications requiring intervention
  - 2—Minor complications requiring intervention
  - 3—No complications but difficulties requiring no intervention
  - 4—No complications, smooth procedure
  - 5—No complications, perfect procedure
5. Final Technical Outcome:
  - 1—Strongly unsatisfactory
  - 2—Somewhat unsatisfactory
  - 3—Neutral
  - 4—Somewhat satisfactory
  - 5—Strongly satisfactory
6. Overall Satisfaction with Surgical Result:
  - 1—Strongly unsatisfied
  - 2—Somewhat unsatisfied
  - 3—Neutral
  - 4—Somewhat satisfied
  - 5—Strongly satisfied
